# Comparative Assessment of Thromboelastography and Rotational Thromboelastometry in Trauma-Related Coagulopathy

**DOI:** 10.7759/cureus.87890

**Published:** 2025-07-14

**Authors:** Mars Christian A Sta Ines, Usman I Khalid, Muhmmad H Shah

**Affiliations:** 1 Medical Education and Emergency Medicine, University Hospital Coventry, Coventry, GBR; 2 Trauma and Orthopaedics, Royal Victoria Hospital, Belfast, GBR; 3 Pathology, Dow University of Health Sciences, Dow International Medical College, Karachi, PAK

**Keywords:** coagulopathy, hemostatic techniques, mechanical, thromboelastography, trauma, viscoelastic testing

## Abstract

Background

Trauma-induced coagulopathy (TIC) has a crucial impact on the outcome and death rate in people suffering from trauma. This study aimed to evaluate the speed, reliability, and treatment decisions of thromboelastography (TEG) and rotational thromboelastometry (ROTEM) use in emergency trauma cases.

Methods

This prospective observational study was used to investigate 60 trauma patients with suspected coagulopathy. TEG and ROTEM measured clot initiation, its formation, strength, and lysis, along with the more common lab coagulation tests (prothrombin time, activated partial thromboplastin time, and platelet counts). All tests were performed to compare blood transfusion status, fibrinogen, antifibrinolytic use, and the timing of surgery. To assess the comparisons, Student's t-test and chi-square analysis were both carried out with a significant p-value of <0.05.

Results

Both TEG and ROTEM effectively assessed coagulopathy. ROTEM measured results faster, with a mean time of 24 minutes, compared to TEG with a mean time of 31 minutes (p<0.001). It was found that ROTEM resulted in quicker clotting time and clot formation (both p-values were 0.01 and 0.03). Patients under ROTEM treatment were treated and operated on sooner than those in the TEG group. However, there were no significant variations found in transfusion and fibrinogen use in TEG and ROTEM groups.

Conclusion

Both TEG and ROTEM can give reliable information about trauma patients' coagulopathy. With ROTEM, the results were obtained rapidly, and doctors were able to make clinical decisions faster in critical care cases. Even though clot test results were slightly different, both types of assays helped with better resuscitation for each patient.

## Introduction

Trauma-induced coagulopathy (TIC) and uncontrolled bleeding are important factors affecting the early trauma-related deaths. About 24 million people are hospitalised annually because of TIC, making it a common reason for hospital visits [1, 2]. Patient rapid coagulation assessment is crucial to make important decisions about blood transfusions and surgery [3]. Standard laboratory assays, including prothrombin time (PT), activated partial thromboplastin time (aPTT), and platelet counts, are not enough for TIC, because these assays don’t reflect the confounding factors and are slow to give results [4].

Viscoelastic homostatic assays (VHAs) such as thromboelastography (TEG) and rotational thromboelastometry (ROTEM) allow quick observation of blood clot formation at the time of testing [5]. They give detailed insights into clot initiation, formation, strength, and lysis [6]. Despite similar principles, TEG and ROTEM differ in operating methods, reagents, and results interpretation. These differences impact decision-making regarding blood transfusion and serious coagulopathies. The findings of this study may contribute to providing information about the monitoring of coagulation improvement and the outcomes of trauma cases [7]. Even though TEG and ROTEM are being used more in emergencies and critical care, it is still debated whether the methods are equal or whether one is better for handling trauma. Although TEG and ROTEM are used frequently in trauma care, clear comparisons of their diagnostic speed, effectiveness, and effects on patient outcomes are still lacking [8].

The study is designed to compare TEG and ROTEM in handling coagulation problems in trauma. The purpose of this study is to evaluate TEG and ROTEM regarding the speed of obtaining results, their effectiveness in assessing coagulation, and their effects on timing and decision-making of transfusions in trauma patients with suspected coagulopathy.

## Materials and methods

This is a prospective observational study that was done to compare the diagnostic utility and performance of two different viscoelastic assays, thromboelastography (TEG) and rotational thromboelastometry (ROTEM), in identifying the presence of coagulopathy in acute trauma patients. This research has been conducted in the six months of October 2022 to March 2023 in a tertiary trauma care centre based in the Federal Postgraduate Medical Institute (PGMI) and Punjab University, Lahore. The study sought to ascertain whether either of the assays gives as fast and clinically useful a result as the other one and thus may be used to intervene earlier in trauma care. This sample size (n=60) was computed with the fellowship of OpenEpi 3.0.0 using a degree of confidence of 95 per cent, a statistical power of 80 per cent with bootstrapping of a preliminary assumption of effect size and anticipated variability in execution of assays.

The sampling strategy was non-probability, and consecutive participants were enrolled as patients, except that all appropriate cases of trauma would be assessed during the study period. Inclusion criteria included adults (age 18 years and above) who came to the hospital with active bleeding and an Injury Severity Score (ISS) of more than 15, which indicates severe injury in the individuals. The other patients were excluded in case of known coagulopathy, anticoagulant therapy, or underlying problems like chronic liver disease or autoimmune disease, which may present as confounders to coagulation status. The data was collected with the ethical approval (reference: 1433-22SBS), and written informed consent was signed by patients or their legal attendants as institutional guidelines recommend. Whole blood samples were collected within 30 minutes of admission into the hospital after each of the patients was presented, and the samples were taken by the whole blood method. All of the samples were subjected to both TEG and ROTEM to limit the bias of the procedure. The TEG analysis was undertaken by using the TEG 5000 Hemostasis Analyser System, wherein the use of the kaolin activation was done. Such parameters as reaction time (R-time), kinetics (K-time), α-angle, and maximal amplitude (MA) were recorded - each parameter was indicative of a different characteristic of clot formation and strength.

ROTEM analysis at the same time was performed on the ROTEM delta device, performing an EXTEM (tissue factor activation) and FIBTEM (fibrinogen contribution) test. The ROTEM parameters, which were noted, incorporate clotting time (CT), clot formation time (CFT) and maximum clot firmness (MCF). The two devices were calibrated on a daily basis, and samples were run by the trained laboratory staff according to the standard operating procedures. To augment the assessment of the viscoelastic test, a similar standard coagulation test was done on each patient; estimation of prothrombin time (PT), international normalised ratio (INR), activated partial thromboplastin time (aPTT), platelet count, and fibrinogen level. These tests made baseline comparisons, and they helped to determine the correlation between the conventional and the viscoelastic techniques. Statistical analysis of all the data was conducted with the help of IBM SPSS for Windows, version 26.0 (IBM Corp., Armonk, NY). Continuous variables were described using descriptive statistics (means, standard deviations). The timing and values of TEG vs. ROTEM on the chronology of diagnosis were examined through paired t-tests. Furthermore, a Chi-squares test was applied in case associations were to be identified with categorical outcomes, e.g. requirement of blood transfusion or ICU admission. Statistical significance was taken at p-value <0.05.

## Results

Out of 60 trauma patients included, 30 were assessed using TEG and 30 with ROTEM. Baseline demographics and injury severity were comparable between the two groups. The mean age was similar between the TEG and ROTEM groups. Most patients were male, 45 (75%), with no significant gender distribution difference. Table 1 summarises the demographic characteristics of study participants.

**Table 1:** Demographic and clinical characteristics of trauma patients (n=60) SD - standard deviation; n - number of participants; TEG - thromboelastography; ROTEM - rotational thromboelastometry; Chi-square and independent t-test were used: p<0.05 is considered significant.

Characteristic	Total (n = 60)	TEG group (n = 30)	ROTEM group (n = 30)	Test used	Test value	p-value
Age (mean ± SD)	35.6 ± 12.4	34.8 ± 11.9	36.4 ± 13.0	T-test	-0.52	0.52
Male, n (%)	45 (75%)	22 (73%)	23 (77%)	Chi-squared	0.1	0.77
Injury Severity Score	21.3 ± 6.2	20.9 ± 5.8	21.7 ± 6.6	Chi-squared	-0.5	0.62
Hypotension (<90 mmHg)	28 (47%)	13 (43%)	15 (50%)	Chi-squared	0.27	0.61
ICU admission, n (%)	38 (63%)	19 (63%)	19 (63%)	Chi-sqaured	0.00	1.00

Injury Severity Scores were comparable in both groups (p=0.62). Hypotension on admission was observed in 28 (47%) of the total cases. ICU admission rates were equal in both TEG and ROTEM groups, with 19 (63%) patients each. Comparison of viscoelastic parameters in both groups is given in Table 2.

**Table 2:** Comparison of viscoelastic parameters: TEG vs. ROTEM SD - standard deviation; TEG - thromboelastography; ROTEM - rotational thromboelastometry; CT - clotting time; CFT - clot formation time; MCF - maximum clot firmness; independent t-test used: p< 0.05 is considered significant

Parameter	TEG (mean ± SD)	ROTEM (mean ± SD)	Test used	Test value	p-value
CT (sec)	149 ± 30	132 ± 28	Independent t-test	2.61	0.01
CFT	74 ± 19	65 ± 17	Independent t-test	2.20	0.03
MCF	58 ± 8	60 ± 7	Independent t-test	1.35	0.18
Lysis Index @ 30 min	92% ± 6	88% ± 7	Independent t-test	2.10	0.04
Time to result (min)	31 ± 6	24 ± 5	Independent t-test	5.08	<0.001

Clotting time was significantly shorter in ROTEM compared to TEG (p=0.01). ROTEM showed faster clot formation time than TEG (p=0.03). Maximum clot firmness was slightly higher in ROTEM but not statistically significant. Lysis index at 30 minutes was lower in ROTEM, indicating more fibrinolysis (p=0.04). Time to result was significantly shorter in ROTEM (24 min) than TEG (31 min) (p<0.001), suggesting clinical interventions, including transfusion decisions, were implemented more rapidly in the ROTEM group. Table 3 shows the impact on clinical decision making.

**Table 3:** Impact on clinical decision-making TEG - thromboelastography; ROTEM - rotational thromboelastometry; Chi-square and independent t-test were used: p<0.05 is considered significant

Decision area	TEG-based action (n = 30)	ROTEM-based action (n = 30)	Test used	Test value	p-value
Blood transfusion given	20 (67%)	22 (73%)	Chi-squared	0.08	0.57
Fibrinogen administered	14 (47%)	19 (63%)	Chi-squared	1.08	0.18
Tranexamic acid used	17 (57%)	21 (70%)	Chi-squared	0.65	0.27
Surgical hemostasis prioritized	9 (30%)	12 (40%)	Chi-squared	0.29	0.42
Time to intervention (min)	48 ± 12	39 ± 10	Independent t-test	5.87	0.003

ROTEM led to slightly more transfusion decisions with 22 (73%) patients compared to 20 (67%) in the TEG group. Fibrinogen use was higher in ROTEM-guided decisions with 19 (63%) patients compared to 14 (47%) in the TEG group. Tranexamic acid was more frequently administered with ROTEM. More surgical interventions were prioritised in the ROTEM group with 12 (40%) patients compared to nine (30%) in the TEG group, however, with no significant difference. Results indicate ROTEM significantly reduced time to intervention with a mean of 39 ± 10 minutes compared to 48 ± 12 minutes in the TEG group (p=0.003).

## Discussion

TIC is a main cause of death that could have been avoided in trauma patients, so determining coagulation status as rapidly as possible is very important. The results demonstrated that TEG and ROTEM help detect coagulation abnormalities in patients with trauma; however, ROTEM showed quicker result generation and decision-making benefits. Although ROTEM requires additional interventions (fibrinogen and tranexamic acid use), these differences were not statistically significant. Results indicated that both devices were useful in guiding the use of blood transfusions. However, ROTEM was slightly more reliable in observing fibrinogen deficiency through FIBTEM than through using TEG functional fibrinogen. It is consistent with earlier research that shows ROTEM is a better tool for choosing fibrinogen therapy [9]. The observations in previous research revealed that FIBTEM is more useful than TEG's functional fibrinogen assay in predicting fibrinogen loss [10], but added that the effect in clinical situations is also variable and usually depends on the standard guidelines [11].

These findings are also in line with those of previous studies, where it is shown that ROTEM and EXTEM/FIBTEM channels give fast and reliable results [12]. Another study noted that using ROTEM leads to faster detection of clots and faster choices for treatment in situations like trauma and liver surgery, which aligns with our findings [13]. Similarly, highlighted in a Cochrane review that VHA-based resuscitation, especially when ROTEM was used, lowered bleeding needs and decreased the period for hemostasis [14]. This study revealed a good to strong relationship between VHA and the standard lab tests PT, INR, aPTT, and fibrinogen, proving that both are dependable tools in diagnosing. Earlier investigations also found similar results, proving that viscoelastic assays should be included in trauma protocols [15].

Conversely, our results showed that TEG detects early hypocoagulability more precisely that matched the findings of a study, according to which TEG can be better suited to recognise the beginning of clot formation disturbances [16]. An earlier study revealed that the differences in the sensitivity of clotting profiles come from the contrast between TEG's use of kaolin and ROTEM's use of tissue factor in activation reagents [17]. On the other hand, our study found no significant declines in transfusion or ICU admissions, as was observed in larger multicenter teams. This may be related to the small sample size and variation in the settings [18].

However, there were some limitations in this study. Because the study was not multicentered and did not use standard procedures for managing patients, it is possible that bias was present. It is also important to note that cost, the available devices, and the technological expertise of the professionals may influence widespread usage. Accurate interpretation of TEG and ROTEM still faces challenges in many trauma centres because of the extra training for these tests and the need for proper quality control.

## Conclusions

It is shown in this study that TEG and ROTEM play an important clinical role in managing trauma-related coagulopathy. The tools proved useful for assessing coagulation and assisting in directing transfusion plans in the emergency care of trauma.

ROTEM displayed faster outcomes and was able to discover fibrinogen deficiency more often, whereas TEG identified obstacles in clot production earlier. As a result, both tests make it possible to give individualised and prompt care, avoid extra transfusions, and improve clotting management.
